# HIV Drug Resistance-Associated Mutations in Antiretroviral Naïve HIV-1-Infected Latin American Children

**DOI:** 10.1155/2010/407476

**Published:** 2011-01-18

**Authors:** Luis E. Soto-Ramirez, Roberto Rodriguez-Diaz, D. Robert Harris, Rohan Hazra

**Affiliations:** ^1^Department of Infectious Diseases, Instituto Nacional de Ciencias Médicas y Nutricion, 14000 Ciudad de México, DF, Mexico; ^2^Westat, Rockville, MD 20850-3129, USA; ^3^Pediatric, Adolescent and Maternal AIDS Branch, Eunice Kennedy Shriver National Institute of Child Health and Human Development (NICHD), National Institutes of Health, 6100 Executive Boulevard, Room 4B11, Bethesda, MD 20892-7510, USA

## Abstract

Our goal was to describe the presence of HIV drug resistance among HIV-1-infected, antiretroviral (ARV) naïve children and adolescents in Latin America and to examine resistance in these children in relation to drug exposure in the mother. Genotyping was performed on plasma samples obtained at baseline from HIV-1-infected participants in a prospective cohort study in Brazil, Argentina, and Mexico (NISDI Pediatric Study). Of 713 HIV-infected children enrolled, 69 were ARV naïve and eligible for the analysis. At enrollment, mean age was 7.3 years; 81.2% were infected with HIV perinatally. Drug resistance mutations (DRMs) were detected in 6 (8.7%; 95% confidence interval 3.1–18.2%) ARV-naïve subjects; none of the mothers of these 6 received ARVs during their pregnancies and none of the children received ARV prophylaxis. Reverse transcriptase mutations K70R and K70E were detected in 3 and 2 subjects, respectively; protease mutation I50 V was detected in 1 subject. Three of the 6 children with DRMs initiated ARV therapy during followup, with a good response in 2. The overall rate of primary drug resistance in this pediatric HIV-infected population was low, and no subjects had more than 1 DRM. Mutations associated with resistance to nucleoside reverse transcriptase inhibitors were the most prevalent.

## 1. Introduction

Primary HIV-1 drug resistance has been reported in up to 20% of HIV-infected adults in the United States and Europe [1–3] and has led to recommendations that all treatment-naïve adults undergo resistance testing when they enter care [4]. Data on primary (or initial) resistance in HIV-infected infants and children are more limited but have led to similar recommendations [5]. Recent studies of infected infants in the United States have demonstrated rates of primary resistance to at least one antiretroviral (ARV) in the range of 19–24%, with most being mutations associated with nonnucleoside reverse transcriptase inhibitor (NNRTI) resistance [6, 7]. Rates reported from older children in the United Kingdom are lower (3 of 44, 6.8%) [8]. In a study from Brazil, primary resistance was seen in 7 of 26 (27%) perinatally infected children [9], and a study from Argentina demonstrated primary resistance in 5 of 22 (23%) infected infants but much lower rates (1 of 45, 2.2%) in infected children 1–14 years of age [10]. When examined in some of these studies, maternal ARV history did not generally provide a clear explanation for the results seen in the infants and children. 

The primary objectives of this analysis were to describe the presence of HIV drug resistance mutations (DRMs) in ARV treatment-naïve, HIV-1-infected children enrolled in an observational study conducted in multiple sites in Latin America and to examine subsequent treatment history and response. 

## 2. Material and Methods

In 2002, The Eunice Kennedy Shriver National Institute of Child Health and Human Development (NICHD) began enrollment to the NICHD International Site Development Initiative (NISDI) pediatric protocol at 15 sites in Brazil, Mexico, and Argentina [11]. The NICHD IRB, the data management and statistical center IRB, separate in-country ethics committees, and national review boards (where required, i.e., Brazil) reviewed and approved the protocol. Informed consent was obtained from either parents or guardians or from subjects who were able to provide consent based upon local laws. Assent was obtained from subjects >8 years of age when developmentally appropriate.

The following assessments were performed at 6-month intervals as part of the NISDI pediatric protocol: medical history, physical examination, hematology, flow cytometry, and HIV viral load testing. Blood samples were collected at enrollment and yearly thereafter and sent to a central repository for storage. The study population for this investigation was comprised of all ARV-naïve, HIV-1-infected children who were enrolled in the NISDI Pediatric Protocol as of February 1, 2005. Children born to mothers with known or suspected HIV infection were considered to be perinatally exposed. Children who had received ARVs during the first 49 days of life for prophylaxis for mother-to-child transmission (MTCT) of HIV were considered ARV naïve for purposes of this investigation. 

Plasma samples were assayed with the ViroSeq HIV-1 genotyping system v 2.6, Celera Diagnostics, at the Molecular Virology Laboratory of the Instituto Nacional de Ciencias Médicas y Nutricion Salvador Zubiran in Mexico City, according to the manufacturer's specifications. Reportable DRMs were based on definitions of surveillance DRMs from an international consensus document [12].

Characteristics of the study population are described using simple descriptive statistics. The association of DRMs with categorical characteristics was evaluated using the Fisher's exact test. For continuous scaled measures, the Student's *t*-test or Wilcoxon Rank Sums test (nonparametric test) was used to assess differences between groups. Because only a small number of DRMs was detected, no attempt was made to model the risk of DRMs as a function of subject characteristics. All analyses were performed using SAS, Version 9.1. 

## 3. Results

Of the 713 HIV-infected children and adolescents enrolled in the NISDI Pediatric Protocol at clinical sites in Argentina, Brazil, and Mexico as of February 1, 2005, a total of 85 subjects were ARV naïve and eligible for inclusion in this study. Sixteen were excluded because they either did not have a sample in the repository or viral DNA could not be amplified from their specimen, leaving 69 subjects eligible for the final analysis. 

Characteristics of the overall study population are shown in Table 1. Most were perinatally HIV infected, and only 12.5% of the HIV-infected mothers of the perinatally infected subjects had received ARVs during pregnancy or at labor and delivery. DRMs were detected in samples from 6 of the 69 eligible subjects (8.7%; 95% confidence interval [CI] 3.1–18.2%). 

Results from examining the association of DRMs with characteristics of the study population (Table 1) indicate that there were no differences in the presence or absence of DRMs according to subject's country of origin, age, gender, or mode of HIV transmission (*P* ≥ .6). Among perinatally exposed subjects, mother's receipt of ARVs during pregnancy or at labor and delivery was not associated with whether or not DRMs were detected (*P* = .55). The presence or absence of DRMs was not associated with CD4 percent, CD4 count, CDC clinical classification, or immunological category (*P* > .09). The occurrence of DRMs was not associated with HIV-1 RNA at or nearest the time when the resistance testing specimen was collected; the geometric mean HIV-1 RNA measure for those with and without DRMs detected was 24,592 and 46,806, respectively (*P* = .42). There was only one death among study subjects; DRMs were not detected in this subject's specimen.

The subjects with DRMs identified ranged in age at time of enrollment from 4 to 6 years of age and 50% were female. All 6 subjects were perinatally HIV infected with subtype B virus. None of the mothers of these 6 were known to have received ARVs during pregnancy, and none of these 6 subjects received ARVs during the first 49 days of life. There were 3 different mutations detected among the 6 subjects with DRMs: reverse transcriptase mutations K70R (detected in 3 subjects), K70E (detected in 2 subjects), and protease mutation I50 V (detected in a single subject). None of the subjects had more than one DRM detected in their enrollment sample and none had DRMs associated with resistance to nonnucleoside reverse transcriptase inhibitors detected. Four of the six subjects (66.7%) were classified as having no evidence of immune suppression. 

Three of the 6 subjects with DRMs started ARV therapy while being followed on the study. One subject started zidovudine/lamivudine/efavirenz, and data at approximately 9 months after initiation of ARVs showed a higher viral load and CD4 cell count continuing to decrease (data not shown). Two other subjects started zidovudine/lamivudine/efavirenz and zidovudine/lamivudine/nelfinavir respectively, and both achieved virologic suppression. 

## 4. Discussion

Among this population of HIV-1-infected children and adolescents from Latin America who were ARV naïve at time of enrollment with relatively low plasma viral loads and relatively high CD4+ counts, DRMs were detected in repository samples from 6 of 69 subjects (8.7%, 95% CI 3.1–18.2%). The population we studied is likely representative of the pediatric HIV population at these sites with expertise in pediatric HIV and availability of ARVs, but we cannot comment on how representative our population is of the whole region. In comparison to other studies, this rate of primary drug resistance is lower than that reported in children in Northeast Brazil [9] and higher than the 0% rate reported in 24 children in Sao Paulo, Brazil [13] (although reverse transcriptase mutation K219N was seen in one subject in the latter study, which, by our definition [12], yields a rate of 4.2%). Studies from the United States and Argentina have shown higher rates in HIV-infected infants [6, 7, 10]. However, all of these studies are quite small and larger cohorts and datasets are necessary to document these rates around the world and to examine changes in them as access to ARVs expands. In addition, in several of these other studies and in ours, specimen collection was performed long enough after infection that some DRMs may have become undetectable, thus underestimating the true rate of resistance and also helping to explain the wide range of results seen.

The DRM identified in 5 of the 6 subjects in our study with primary resistance was associated with nucleoside reverse transcriptase inhibitor resistance; the 6th subject had a protease inhibitor-related DRM. None of the mothers of these 6 children were known to have received ARVs during pregnancy, and none of the 6 received ARV prophylaxis, suggesting that the mothers were infected with the primary resistant strain and transmitted this to their babies. A limitation of our study is that we do not have samples from the mothers to be able to assess this.

During the course of followup, 3 of the 6 subjects with DRMs in our study began highly active ARV therapy, with 2 demonstrating a good response. While these numbers are obviously too small to be able to draw any conclusions, more such data on treatment outcomes in pediatric patients with primary drug resistance are needed as ARV treatment programs expand around the world.

## Figures and Tables

**Table 1:** Characteristics of study population overall and according to whether or not DRMs were detected.

Characteristic	Overall	DRMs detected	No DRMs detected	*P* value*
Number of subjects	69	6 (8.7%)	63	

Country of origin:				
Argentina	5 (7.2%)	0 (0.0%)	5	0.74
Brazil	55 (79.7%)	6 (10.9%)	49	
Mexico	9 (13.0%)	0 (0.0%)	9	

Age at enrollment (years):				
Mean (SD)	7.3 (6.0)	5.2 (0.8)	7.5 (6.2)	0.64
Median	6.0	5.0	6.0	

Gender:				
Female	40 (58.0%)	3 (7.5%)	37	0.69
Male	29 (42.0%)	3 (10.3%)	26	

Subject's risk for HIV infection:				
Blood product transfusion	1 (1.5%)	0 (0.0%)	1	1.00
Consensual sexual contact	8 (11.6%)	0 (0.0%)	8	
Perinatal exposure	56 (81.2%)	6 (10.7%)	50	
Unknown	4 (5.8%)	0 (0.0%)	4	

Among perinatally infected, did mother take ARVs during pregnancy or at labor and delivery?				
Yes	7 (12.5%)	0 (0.0%)	7	0.55
No	44 (78.6%)	5 (11.4%)	39	
Unknown	5 (8.9%)	1 (1.8%)	4	

CD4 percent at or nearest to the time when the resistance testing specimen was collected:				
Mean (SD)	24.2 (10.9)	25.2 (8.6)	24.1 (11.2)	0.42
Median	24.0	28.0	23.0	
<15%	11 (17.5%)	1 (9.1%)	10	0.64
15–24.9%	23 (36.5%)	1 (4.3%)	22	
≥25%	29 (46.0%)	4 (13.8%)	25	
Missing	6	0	6	

CD4 absolute count (cells/mm^3^) at or nearest to the time when the resistance testing specimen was collected:				
Mean (SD)	779 (615)	769 (354)	780 (637)	0.65
Median	668	678	668	
<200 cells/mm^3^	9 (13.2%)	0 (0.0%)	9	0.84
200–500	16 (23.5%)	1 (6.3%)	15	
>500	43 (63.2%)	5 (11.6%)	38	
Missing	1	0	1	

CDC clinical classification:				
Category N	18 (26.5%)	4 (22.2%)	14	0.09
Category A	9 (13.2%)	1 (11.1%)	8	
Category B	4 (5.9%)	0 (0.0%)	4	
Category C	37 (54.4%)	1 (2.7%)	36	
Missing	1	0	1	

HIV-1 viral load (copies/mL) at or nearest to the time when the resistance testing specimen was collected:				
Mean	214,448	34,062	232,191	0.25
SD	522,665	31,248	544,831	
Median	40,100	25,521	44,000	
Vital status of study subject:				
Dead	1 (1.4%)	0 (0.0%)	1	1.00
Alive	68 (98.6%)	6 (8.8%)	62	

*The significance of associations of categorical characteristics with DRMs was determined based on Fisher's Exact test, while the association with continuous scaled characteristics was based on the Student's *t*-test (log_10_-transformed viral load) or nonparametric testing (Wilcoxon test).
